# IFNγ blockade in capillary leak site improves tumour chemotherapy by inhibiting lactate-induced endocytosis of vascular endothelial-cadherins

**DOI:** 10.7150/ijbs.78248

**Published:** 2023-02-27

**Authors:** Ruirui Wang, Chen Ni, Xiaohan Lou, Lijing Zhang, Linlin Wang, Xiaohan Yao, Xixi Duan, Jiajia Wan, Pan Li, Zhihai Qin

**Affiliations:** 1 Medical Research Center, the First Affiliated Hospital of Zhengzhou University, Zhengzhou, Henan, 450052, China.; 2 Institute of Biophysics, Chinese Academy of Sciences, Beijing, 100101, China.

**Keywords:** IFNγ blockade, vascular endothelial-cadherin, lactate, capillary leak, chemotherapy.

## Abstract

IFNγ has long been recognised as a key mediator of tumour immunity and angiostasis. However, IFNγ modulation for cancer therapy is still unsuccessful due to its complex effects on various host cells. In this study, we found that treatment of Lewis lung carcinoma transplants with cisplatin often caused IFNγ-dependent tumour vascular damage. IFNγ induced endothelial glycolysis and lactate production, leading to enhanced endocytosis of vascular endothelial (VE)-cadherin and vessel leakage. We have also developed anti-IFNγ nanoparticles coated with a clot-binding peptide CREKA (CREKA-lipo-anti-IFNγ), which targets the fibrin-fibronectin complex that appears in the leaky site of damaged tumour blood vessels. Blocking IFNγ activity in the leakage site of capillaries using nanoparticles rescued VE-cadherin distribution on the endothelial cellular surface, promoted blood vessel integrity, and improved drug delivery. In conclusion, IFNγ blockade in capillary leak site protected tumour blood vessels from lactate-dependent VE-cadherin loss and enhanced drug delivery during chemotherapy, which provides a basis for tissue-specific IFNγ blockade for tumour therapy.

## Introduction

Efficient drug penetration into solid tumours is a considerable challenge in cancer chemotherapy, which is one of the most important methods of clinical cancer treatment. For example, cisplatin [cis-diamminedichloridoplatinum (II)], a platinum-based anticancer chemotherapeutic drug, has long been used as a first-line drug in the treatment of lung cancer. Unfortunately, in most cases, chemotherapy only delays the development of the disease and has a 40-50% remission rate 1, 2. Many factors influence the response of tumours to chemotherapeutic drugs, including genetic mutations, gene amplification, or epigenetic changes that affect single-cell uptake, metabolism, or export of drugs 3. The tumour vascular network is instinctively leaky compared to physiological vasculatures, which forms the primary barrier for adequate drug penetration 4. Disoriented and hypo-perfused tumour vessels prevent the efficient diffusion of chemotherapeutic drugs, while retard the anti-tumour efficacy of immune cells. The immature and “leaky” tumour vasculature might also lead to cancer cell intravasation, increasing the metastatic potential. Therefore, improvement of tumour vascular integrity or “vascular normalization” has emerged as a new treatment goal, aiming to provide a mature tumour vasculature, with higher perfusion, decreased cancer cell extravasation, and higher efficacy for anti-cancer therapies 5. Chemotherapeutic drugs and associated inflammatory cytokines can cause capillary leakage 6. Tumour vascular destruction by chemotherapeutic agents may further impair drug delivery 7. Noteworthily, this also allows the release of plasma proteins into the tumour stroma that provides potential drug targets for the repair of vascular damage 8, 9. Plasma fibrinogen enters the tumour interstitial space at the capillary leaky site, where it is transformed to fibrin by tissue procoagulant factors and cross-links with stromal proteins, such as fibronectin 8. Fibrin-fibronectin complexes that appear in leaky tumour blood vessels can be targeted by CREKA, a clot-binding peptide 8, 9. Nevertheless, mechanisms whereby chemotherapeutic drugs cause capillary leaks and strategies to interfere them for sufficient drug penetration are not fully understood.

The integrity of blood vessels is regulated by endothelial metabolism 10, 11. The complete vascular structure consists of endothelial cells, basement membranes, and pericytes 12. Endothelial cells are interconnected by adherent junctions 12. Vascular endothelial cadherin (VE-cadherin) is responsible for endothelial adherent junction assembly and plays a key role in the maintenance of vascular integrity 13. Endothelial cell metabolism largely depends on glycolysis, which may be dysregulated in diseases 14. Inhibition of glycolysis in the endothelium is a possible way of enhancing vascular integrity by reducing endocytosis of VE-cadherins and promoting drug delivery during chemotherapy 4. Inflammatory cytokines are important regulators of endothelial metabolism. We previously reported that IL-17A stimulates endothelial fatty acid β-oxidation 15. Lee et al. have demonstrated that IFNγ impairs endothelial glucose metabolism and results in a metabolic shift toward increased fatty acid oxidation in human coronary artery endothelial cells 16. Thus, inflammatory cytokines are potential targets for modulating endothelial integrity by regulating endothelial metabolism.

IFNγ is an important modulator of tumour vascular functions 17-19. It is a pleiotropic inflammatory factor that has long been considered as an important effector molecule in anti-tumour immunity 20. IFNγ-mediated angiostasis is an important mechanism for T cell-mediated rejection of tumours 17, 21. In the process of cyclopamine-induced tumour rejection, macrophages are activated to produce IFNγ and inhibit angiogenesis 22. In contrast, IFNγ has been shown to promote the outgrowth of tumour cells with an immune evasive phenotype 23, 24. IFNγ combined chemotherapy has been used in early studies to treat tumours; however, IFNγ led to an increase in the lethal rate of cancer patients 23, 24. Recently, we found that IFNγ has a modulating effect on the integrity of brain endothelial cells under inflammatory conditions 25. Our recent study has also shown that IFNγ reduces the expression of genes involved in DLL4 signalling pathways in endothelial cells and lowers the expression of adhesion protein N-cadherin on perivascular cells, thereby stripping them from vessels 19, 26. Therefore, we presumed an opportunity for targeting IFNγ to improve vascular function and promote drug delivery for tumour chemotherapy.

In this study, we found that cisplatin-induced tumour vascular damage was largely dependent on IFNγ. IFNγ stimulation enhanced endothelial glycolysis and increased lactate production, which promoted endocytosis and degradation of VE-cadherin, leading to vascular leakage. We developed anti-IFNγ nanoparticles coated with CREKA peptide for the specific delivery of anti-IFNγ antibody to capillary leaky sites. The resulting nanoparticles, CREKA-lipo-anti-IFNγ, successfully rescued VE-cadherin distribution on endothelial cells and promoted chemotherapy. Our results provide a basis for improving tumour chemotherapy through tissue-specific IFNγ blockade.

## Methods

### Cell culture

The mouse Lewis lung carcinoma (LLC) cell line and the mouse endothelial cell line sEND.1 cell line were cultured in a medium consisting of DMEM (#11995; Gibco) supplemented with 10% foetal bovine serum (FBS; PAA).

### Mouse model

Female C57BL/6 wild-type (WT) mice were purchased from Vital River Laboratories (Beijing, China). LLC cells (1 × 10^6^) in 100 μL were subcutaneously (s.c.) injected into 6 to 8-week-old C57BL/6 female mice. Tumour growth was monitored every other day, and mortality was recorded daily during the entire experimental period. The lung cancer spontaneous mouse model (TetO-EGFR^L858R^; CCSP-rtTA) was a gift from Professor Xi Lin, Tsing Hua University. Mice were treated as previously described 27. All mice were housed in a specific pathogen-free facility at Zhengzhou University. Animal protocols were approved by the Animal Care and User Committee of the First Affiliated Hospital of Zhengzhou University (2021-KY-0626-002).

### *In vivo* Treatments

Cisplatin (Cpt, 1 mg/kg, P4394, Sigma) and anti-IFNγ prepared from hybridoma ATCC HB-170 as previously described 26, 28 (clone R46-A2; 2.5 mg/kg), were intraperitoneally (i.p.) injected into the mice (6-8 weeks old). CREKA-lipo-anti-IFNγ (2.5 mg/kg) was administered by tail vein injection. Cisplatin was injected when the tumour volume reached ~200 mm^3^ every alternate day, four times in total. Anti-IFNγ or CREKA-lipo-anti-IFNγ was administered two days after the first injection of cisplatin. To assess chemotherapy drug delivery, a single dose of cisplatin (10 mg/kg, P4394, Sigma) was injected i.p 6-8 h prior to euthanasia.

### Histopathological and immunostaining

Tumour tissues were harvested and embedded in optimal cutting temperature compound (OCT compound). They were then frozen and cut using a cryostat. For histological staining of tumour tissues, frozen sections (7 μm) were stained with Rat-anti-CD31 (1:200, BD Biosciences Cat# 550274, RRID: AB_393571), Rabbit-anti-CD31 (1:100, Abcam Cat# ab28364, RRID: AB_726362), Goat-anti-VE-cadherin (1:200, R&D Systems Cat# AF1002, RRID: AB_2077789), Rabbit-anti-NG2 (1:200, Millipore Cat# AB5320, RRID: AB_11213678), Rat-anti-CD146 (1:100, Biolegend Cat#134701), Rabbit-anti-GLUT1 (1:200, Millipore Cat# 07-1401, RRID: AB_1587074), Rat-anti-cisplatin-modified DNA (1:200, MABE416, Millipore), and Rabbit-anti-γ-H2AX (1:200, Cell Signalling Technology Cat# 9718, RRID: AB_2118009). Pictures were acquired using a Vectra, Perkin Elmer.

### Image analysis

For quantification of CD31^+^ blood vessels in tumour, the area of CD31^+^ blood vessels in each high-power field of tumour was analysed using Vectra, and the density of CD31^+^ blood vessels were presented as CD31^+^ area / total sectional area × 100%. Images used for analysis were derived from 2 to 3 tumours.

For quantification of VE-cadherin^+^ blood vessels in tumour, colocalization of VE-cadherin and CD31 in each high-power field of tumour was analysed using Vectra, and the density of VE-cadherin^+^CD31^+^ blood vessels were presented as the area of colocalization / CD31^+^ area × 100%. Images used for analysis were derived from 2 to 3 tumours.

For quantification of pericyte coverage in tumour, NG2 (neuron-glial antigen 2) was used as the pericyte marker. NG2 is a marker of glial progenitor cells in the central nervous system, and has become the most common marker for pericytes. Pericyte specific deletion of NG2 leads to decreased pericyte ensheathment of endothelial cells, reduced vessel patency, and increased vessel leakiness 29, 30. Colocalization of NG2 and CD31 in each high-power field of tumour was analysed using Vectra, and pericyte coverage was presented as the area of colocalization / CD31^+^ area × 100%. Images used for analysis were derived from 3 tumours.

For quantification of GLUT1^+^ area in tumour, the area of GLUT1^+^ cells in each high-power field of tumour was analysed using Vectra, and GLUT1^+^ area was presented as GLUT1^+^ area/ total sectional area × 100%. Images used for analysis were derived from 3 tumours.

For quantification of Cpt^+^ cells in tumour after treatment, the number of Cpt^+^ cells in each high-power field of tumour was counted using Vectra, and the number of Cpt^+^ cells was presented as the number of Cpt^+^ cells / the number of DAPI^+^ cells × 100%. The quantification of γ-H2AX^+^ cells was similar to the quantification of Cpt^+^ cells. Images used for analysis were derived from 2 to 3 tumours.

### Blood vessel perfusion

Fluorescein isothiocyanate (FITC)-lectin (0.5 mg/mL, Vector Laboratories Cat# FL-1171, RRID: AB_2307440) was injected intravenously into tumour-bearing mice. Ten minutes later, mice were perfused by intracardiac injection of 1% paraformaldehyde (PFA) to remove circulating lectin, and tumour tissues were collected and embedded in OCT. Tumour sections (7 μm) were stained with Rat-anti-CD31 (1:200, BD Biosciences Cat# 550274, RRID: AB_393571) followed by an Alexa-Fluor-555 Goat-anti-Rat second antibody (1:400; Molecular Probes Cat# A-21434, RRID: AB_141733). Perfused blood vessels were calculated as FITC-lectin^+^CD31^+^ area/ CD31^+^area × 100%.

### Dextran leakage assay

FITC-dextran (25 mg/mL, 46945, 70 kDa, Sigma-Aldrich, Bornem, Belgium) was administered intravenously to tumour-bearing mice. Mice were perfused by intracardiac injection of 1% PFA to remove circulating dextran ten minutes after injection, and tumours were harvested. The percentage of leaky vessels was measured as FITC-dextran area/total sectional area × 100%.

### RNA extraction and quantitative real-time polymerase chain reaction (qPCR)

To detect the mRNA expression of inflammatory factors in tumour tissues, total RNA was extracted from tumour tissues using TRIzol reagent (9109, Takara) according to the manufacturer's instructions. First-strand cDNA was synthesised from 1 µg of total RNA using the Prime Script™ RT reagent kit (RR047A, Takara). qPCR was performed using SYBR Premix Ex Taq II (RR820A, Takara) and assessed using an Agilent Mx3005P instrument. The abundance of mRNA for each gene of interest was normalised to that of GAPDH. The primers used for the qPCR as follows: *IL-1β* forward: TTCAGGCAGGCAGTATCACTC, reverse: GAAGGTCCACGGGAAAGACAC; *IFNγ* forward: ATGAACGCTACACACTGCATC, reverse: CCATCCTTTTGCCAGTTCCTC;* TNFα* forward: GACGTGGAACTGGCAGAAGAG, reverse: TTGGTGGTTTGTGAGTGTGAG;* IL-6* forward: TAGTCCTTCCTACCCCAATTTCC, reverse: TTGGTCCTTAGCCACTCCTTC;* IL-10* forward: GGTTGCCAAGCCTTATCGGA, reverse: ACCTGCTCCACTGCCTTGCT;* IL-17* forward: TTTAACTCCCTTGGCGCAAAA, reverse: CTTTCCCTCCGCATTGACAC.

### Isolation of tumour endothelial cells and RNA sequencing

Mouse endothelial cells were isolated from tumours by using Tumor Dissociation Kit (Miltenyi, #130-096-730) and Tumor Cell Isolation Kit (Miltenyi, #130-110-187) according to the instructions, and then sorted by Flow cytometry by staning CD31. Isolated tumour endothelial cells were used for RNA sequencing. The data was analyzed by Z-Score normalization.

### Synthesis of CREKA-lipo

DSPE-PEG-MAL (3,800 Da) (8.5 mg) and CREKA (647 Da) peptide (1.78 mg) were dissolved in 4 mL of 10% methanol purged with nitrogen and stirred for 4 h at room temperature. The resulting product was dialysed against water for 24 h and then freeze-dried to obtain DSPE-PEG-CREKA and used as CREKA-lipo 31.

### Preparation and characterization of liposome carrying anti-IFNγ antibody

Liposomes loaded with anti-IFNγ antibody (CREKA-lipo-anti-IFNγ) were prepared using the double emulsion-solvent evaporation method 32. Briefly, an aqueous solution of anti-IFNγ antibody (0.5 mg) in 15 µL of water was emulsified in 0.2 mL of chloroform containing 0.5 mg cholesterol, 15 mg dioleoylphosphatidylcholine (DOPC), and 0.5 mg DSPE-PEG-CREKA by sonication for 60 s in an ice bath. This primary emulsion was further emulsified by sonication (70 W for 60 s) in 3 mL of water in an ice bath. The solvent mixture was concentrated under reduced pressure using a rotary evaporator to a volume of 1 mL.

### Characterization of CREKA-Lipo-anti-IFNγ and CREKA-Lipo

The size distribution and zeta potential of CREKA-Lipo**-**anti-IFNγ and CREKA-Lipo were assessed by dynamic light scattering (DLS) using the ZetaSizer Nano series Nano-ZS (Malvern Instruments Ltd., Malvern, UK).

### The drug release profiles of CREKA-Lipo-anti-IFNγ

In order to detect the drug release profiles, CREKA-Lipo**-**anti-IFNγ was maintained in dialysis bag (molecular weight cutoff of 300 kD), and placed in 15 mL PBS (phosphate buffered saline) buffer in a 50 mL tube, general shaking at 37ºC. At certain time points, 1 mL external buffer was taken and amount of anti-IFNγ was detected by UV-spectrophotometer at 280 nm. At the same time, 1 mL fresh PBS was added into the tube to maintain a consistent total volume. The accumulative release of anti-IFNγ was calculated.

### Biosafety evaluation of CREKA-lipo-anti-IFNγ nanoparticles

Mice (6-8 weeks old) were intravenously injected with CREKA-lipo-anti-IFNγ (2.5 mg/kg) or CREKA-lipo. Eight days after treatment, blood serum was collected from the orbital sinus, and concentrations of aspartate aminotransferase (AST), alanine aminotransferase (ALT) (associated with liver function), and creatinine (CR) (associated with kidney function) were measured using the AST/ALT/CR Detection Kit (Servicebio) following the manufacturer's guidance. The animals were sacrificed, and the mouse heart, liver, spleen, lung, and kidney were collected from each mouse for Hematoxylin and Eosin (H&E) staining to evaluate their morphological changes.

### Glycolytic function assay

sEND.1 cells (3,000 per well) dispensed in 80 µL culture medium per well were seeded into XF96 Cell Culture Microplates (Agilent). After overnight culture, cells were treated with or without IFNγ (100 ng/mL, 315-05, Peprotech) and AZD5363 (10 μM, HY-15431, MCE) for 6 h. The culture medium was changed to XF Base Medium (Agilent) containing 1 mM L-glutamine 1 h before starting the assay. Glucose, oligomycin, and 2-deoxy-D-glucose (2-DG) were subsequently injected into the medium at final concentrations of 10 mM, 1 μM, and 50 mM, respectively. The extracellular acidification rate (ECAR) was measured using a Seahorse XF96 extracellular-flux analyser (Agilent). Finally, we used Hoechst 33342 (C0030, Solarbio, China) to normalise the cell numbers while following the manufacturer's instructions.

### Western blotting

sEND.1 cells were harvested and lysed with RIPA buffer (Solarbio, China) supplemented with 1 μM phenylmethylsulfonyl fluoride (Solarbio, China) and protease inhibitor cocktail (Sigma, USA). Cells were then collected and lysed with RIPA buffer for the western blot assay. The Membrane and Cytosol Protein Extraction Kit (P0033, Beyotime, China) was used to extract the cell membrane and cytosol proteins. Quantified protein lysates were measured with the Protein BCA Assay Kit (23228, Thermo Fisher) according to the manufacturer's instructions. Proteins in lysates were resolved on SDS-PAGE gels, transferred onto polyvinylidene fluoride membranes (ISEEQ00010, Millipore), and immunoblotted with Rabbit-anti-GLUT1 (1:1000, Millipore Cat# 07-1401, RRID: AB_1587074), Rabbit-anti-HK2 (1:1000, Cell Signalling Technology Cat# 2867, RRID:AB_2232946), Rabbit-anti-p-PFKFB2 (1:1000, Cell Signalling Technology Cat# 13064, RRID: AB_2798107), Rabbit-anti-PFKFB2 (1:1000, TA314335, OriGene Technologies), Rabbit-anti-p-AKT (1:1000, Cell Signalling Technology Cat# 4060, RRID: AB_2315049), Mouse-anti-AKT (1:1000, Cell Signalling Technology Cat# 2920, RRID: AB_1147620), Rabbit-anti-p-ERK (1:1000, Cell Signalling Technology Cat# 4370, RRID: AB_2315112), Rabbit-anti-ERK (1:1000, Cell Signalling Technology Cat# 4695, RRID: AB_390779), Rabbit-anti-p-P38 (1:1000, Cell Signalling Technology Cat# 4511, RRID: AB_2139682), Mouse-anti-P38 (1:1000, Cell Signalling Technology Cat# 9228, RRID: AB_490886), Goat-anti-VE-cadherin (0.2 µg/ml, R&D Systems Cat# AF1002, RRID: AB_2077789), and Mouse-anti-β-actin (1:3000, ABclonal Cat# AC004, RRID: AB_2737399). Protein bands were visualised using an eECL Western Blot Kit (CW00495, CwBio). Signal intensities were determined using Quantity One Image Analyser (Bio-Rad, Hercules, California, USA; Quantity One 1-D Analysis Software, RRID: SCR_014280).

### Cell immunofluorescence

sEND.1 cells were cultured in confocal dishes (180504, Cellvis) overnight and treated with or without sodium lactate (10 mM, L7022, Sigma), IFNγ (100 ng/mL, 315-05, Peprotech), Lactate dehydrogenase A (LDHA) inhibitor (GNE140, 5 μM, HY-100742, MCE), and bafilomycin (Baf, 100 nM, 1334, TOCRIS) for 12 h. Cells were then fixed with 4% paraformaldehyde for 15 min and permeabilised with 0.5% Triton X-100 for 5 min. Next, cells were blocked with 3% (w/v) BSA in phosphate buffered saline (PBS) for 30 min at 37 °C and then incubated with Goat-anti-VE-cadherin (0.2 μg/mL, R&D Systems Cat# AF1002, RRID: AB_2077789) and Rabbit-anti-LAMP (1:100, Cell Signalling Technology Cat# 9091, RRID: AB_2687579) for 1 h. Next, these were labelled with Alexa-Fluor-555 donkey-anti-goat second antibody (1:400, Thermo Fisher Scientific Cat# A-21432, RRID: AB_2535853) and Alexa-Fluor-488 donkey anti-rabbit second antibody (1:400, Molecular Probes Cat# A-21206, RRID: AB_2535792) for 50 min at 37 °C, and stained with 4',6-diamidino-2-phenylindole (17985-50, Electron Microscopy Sciences) for 10 min. The images were captured using a spinning-disk confocal microscope.

### Glucose analog uptake

2-[N-(7-Nitrobenz-2-oxa-1,3-diaxol-4-yl)amino]-2-deoxyglucose (2-NBDG) is taken up into cells through glucose transporters and phosphorylated by hexokinase. sEND.1 cells were seeded in a 24 well plate. After overnight culture, cells were treated with or without IFNγ (100 ng/mL, 315-05, Peprotech) and AZD5363 (10 μM, HY-15431, MCE) for 12 h. The culture medium was discarded and cells were washed with PBS (pH 7.4). This was followed by the addition of low-glucose culture media supplemented with 2-NBDG (100 µM, N13195, Life Technologies) and an incubation of 45 min at 37 °C. Next, cells were washed with PBS and trypsin was added, and they were harvested, centrifuged at 1,500 rpm for 5 min at 4 °C, washed twice with ice-cold PBS, and kept on ice. A control sample lacking 2-NBDG was used to set the blank in the flow cytometer and gate parameters for 2-NBDG detection.

### Lactate colorimetric assay

sEND.1 cells were seeded in a 6 well plate. After overnight culture, cells were treated with or without IFNγ (100 ng/mL, 315-05, Peprotech) and AZD5363 (10 μM, HY-15431, MCE) for 12 h. The cells were then collected to detect lactate production using a lactate colorimetric/fluorometric assay kit (k607-100, Biovision, USA) while following the manufacturer's instructions.

### Permeability assay

sEND.1 Cells were seeded onto the upper chamber of the transwell system (3415, Corning). When the cells were confluent in the upper chambers, they were stimulated with or without sodium lactate (10 mM, L7022, Sigma), IFNγ (100 ng/mL, 315-05, Peprotech) and LDHA inhibitor (GNE140, 5 μM, HY-100742, MCE) for 48 h. Thirty minutes before harvest, FITC-dextran (10 mg/mL, 46945, 70 kDa, Sigma-Aldrich, Bornem, Belgium) were added to the upper chamber and incubated at 37 °C. Finally, 100 µL of liquid was aspirated into the lower chamber, and the fluorescence of FITC-dextran was measured using a microplate reader.

### Statistical analysis

Statistical analyses using Nonparametric Mann-Whitney test or two-way ANOVA were performed using GraphPad Prism software (GraphPad Prism, RRID:SCR_002798). Differences were considered significant when P values were less than 0.05. Statistically significant differences are indicated as follows: * *p* < 0.05, ** *p* < 0.01, and *** *p* < 0.001.

## Results

### Cisplatin treatment induces tumour vascular leakage and the increase of IFNγ

**Figure 1:**
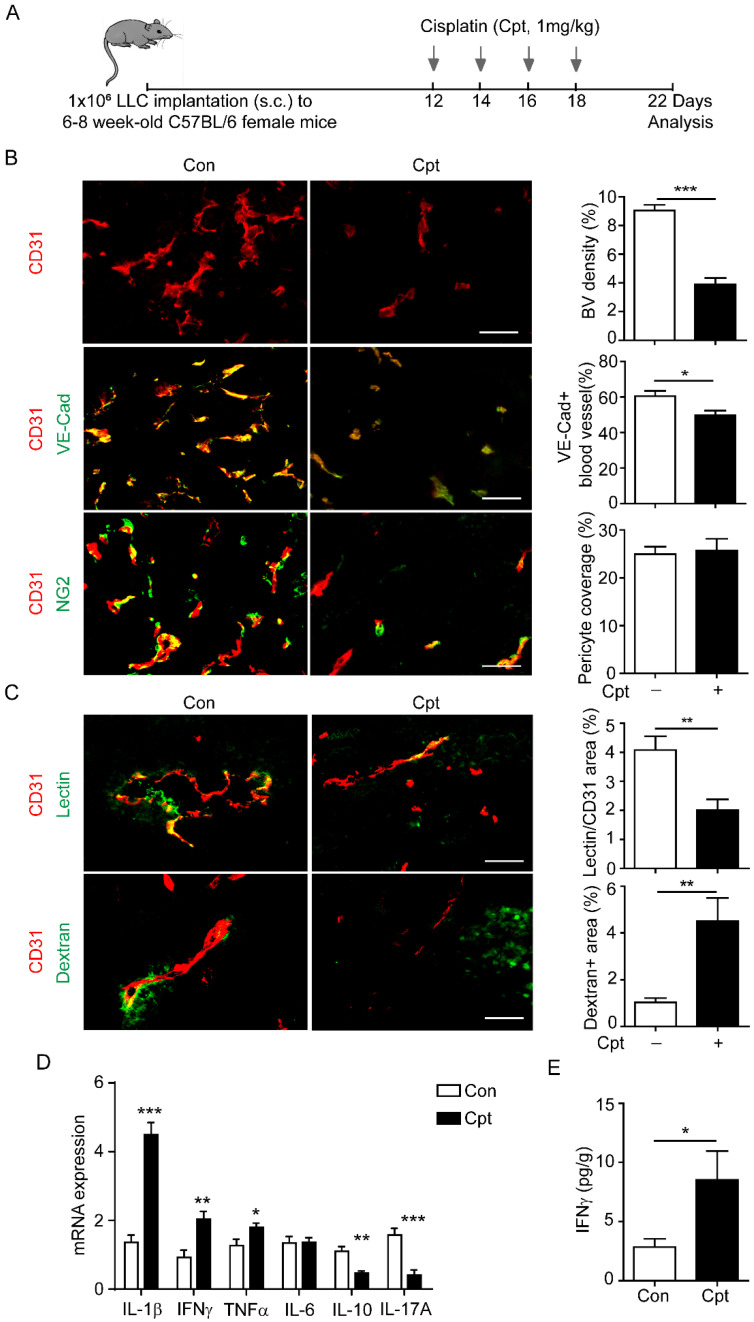
** Cisplatin treatment causes tumour blood vessel leakage and the increase of IFNγ in tumour site.** (**A**) Diagram depicting the generation of Lewis lung carcinoma (LLC) tumour model and schedules for injection and analysis. When tumour volume reached ~200 mm^3^ twelve days after LLC implantation, cisplatin (Cpt, 1mg/kg) was intraperitoneal injected into mice at the indicated time. (**B, C**) Representative images and quantification of vascular density, vascular expression of junctional protein vascular endothelial-cadherin (VE-Cad), pericyte coverage (**B**), vascular perfusion, and permeability (**C**) in tumour vessels with different treatments. Scale bar, 100 μm. (**D**) Analysis of mRNA levels of inflammatory cytokines in tumour tissues two days after the first cisplatin injection. n = 3 for each group. (**E**) Protein levels of IFNγ in tumour tissues from cisplatin- or phosphate buffered saline (PBS)-treated mice. n = 3 for each group. Nonparametric Mann-Whitney test. Values are mean ± SEM. *, *p* < 0.05; **, *p* < 0.01; ***p < 0.001.

A mouse lung cancer model was used to examine the possibility of cisplatin treatment impairing vascular perfusion. LLC cells were subcutaneously transplanted into wild-type (WT) C57Bl/6 mice. Cisplatin (1 mg/kg) was injected every other day, four times, when the tumour volume reached ~200 mm^3^ **(Figure 1A)**. Structural and functional changes in tumour blood vessels were analysed by immunostaining for CD31, VE-cadherin, and NG2 four days after the cessation of chemotherapy (**Figure 1B, C**). In the microcirculation, NG2 identifies perivascular cells along capillaries, and represents the mature and functional blood vessels 29, 30. Cisplatin treatment significantly reduced the density of CD31^+^ tumour blood vessels, the distribution of VE-cadherin in endothelial cells when compared with those in the non-treated tumour, while NG2^+^ pericyte coverage has no difference between the two groups **(Figure 1B)**. These results indicated that cisplatin induced structural damage to the tumour blood vessels and impaired vascular integrity. To measure the vascular perfusion and permeability after cisplatin treatment, FITC-labelled lectin and dextran were injected intravenously into tumour-bearing mice. Cisplatin treatment reduced vascular perfusion, but markedly promoted dextran leakage in the tumour **(Figure 1C)**.

Cisplatin might directly damage endothelial cells or indirectly influence endothelial cells through activation of the immune response 33-35. In a study of lung and breast cancers, the sensitivity of endothelial cells to cisplatin was much lower than that of tumour cells to cisplatin 35. Thus, we suspected that cisplatin-induced vascular injuries were associated with inflammation. We then detected the expression of inflammatory factors in tumour tissues by qPCR after cisplatin treatment. The levels of TNFα, IL-1β, and IFNγ were increased two days after the first cisplatin treatment **(Figure 1D)**. Consistently, the Cytometric Bead Array (CBA) results confirmed that cisplatin induced high expression of IFNγ two days after cisplatin treatment **(Figure 1E)**.

### Neutralising IFNγ blocks cisplatin-induced vascular damages

**Figure 2:**
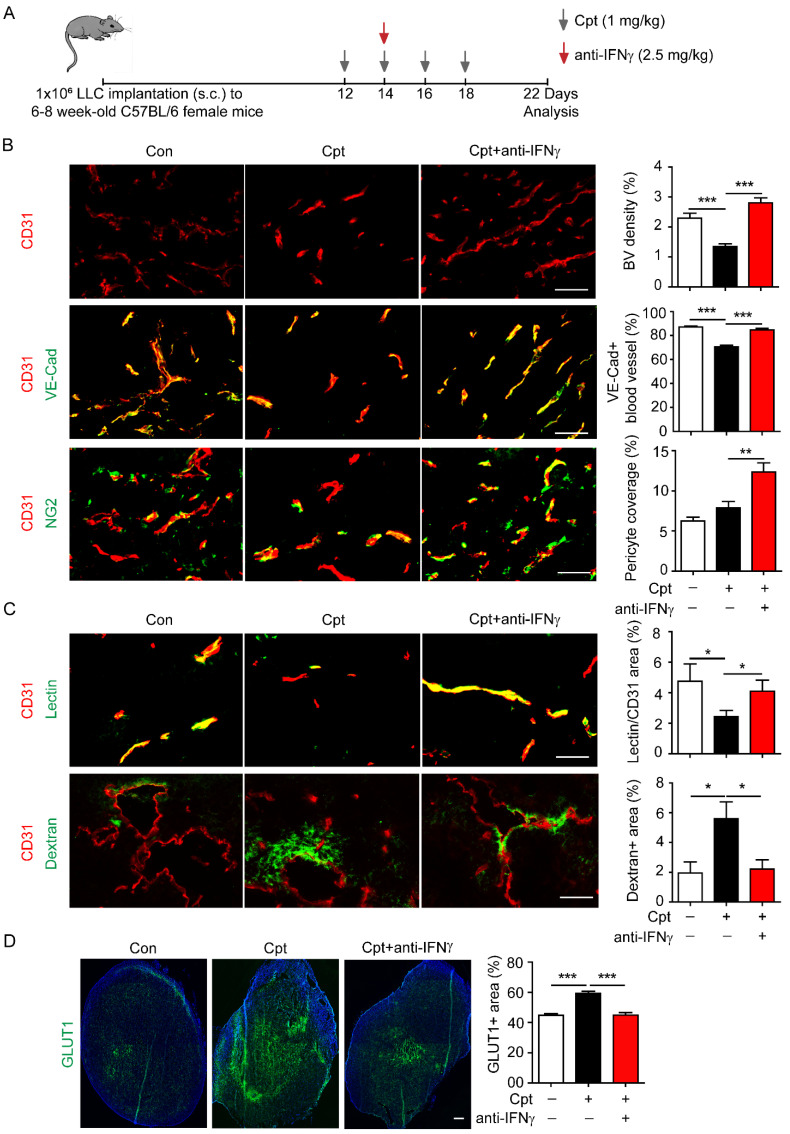
** Neutralizing IFNγ inhibits cisplatin-induced vascular damages.** (**A**) Diagram depicting generation of Lewis lung carcinoma (LLC) tumour model and treatment schedule. (**B**) Representative images and quantifications of vascular density, vascular expression of junctional protein vascular endothelial-cadherin (VE-Cad), pericyte coverage in tumour tissues with different treatments. Scale bar, 100 μm. (**C**) Representative images and quantifications of vascular perfusion and permeability in tumour tissues with different treatments. Scale bar, 100 μm. (**D**) Representative images and quantifications of hypoxia areas (indicated by GLUT1-positive staining) in tumours with different treatments. Scale bar, 1.33 mm. Nonparametric Mann-Whitney test. Values are mean ± SEM. *, *p* < 0.05; **, *p* < 0.01; ***p < 0.001.

Next, IFNγ was neutralised to explore whether the cisplatin-induced vascular damage was dependent on IFNγ. The IFNγ neutralising antibody was administered two days after the first cisplatin injection in the transplanted LLC tumour models **(Figure 2A)**. Based on the observation of ~10 pg/g IFNγ in the tumour tissues (**Figure 1E),** and our own experiences in usage of anti-IFNγ antibodies (clone: R46A2) 17, 36, we applied anti-IFNγ antibody at 2.5 mg/kg. Anti-IFNγ blocked cisplatin-induced reduction in CD31^+^ tumour blood vascular density **(Figure 2B)** and the distribution of VE-cadherin on tumour vessels **(Figure 2B)**. Although cisplatin treatment did not reduce the NG2^+^ pericyte coverage compared with control, anti-IFNγ increased NG2^+^ pericyte coverage compared with cisplatin-treated tumours **(Figure 2B)**. Importantly, anti-IFNγ combined with cisplatin treatment increased the perfusion of lectin into the tumour vessels** (Figure 2C)** while reducing the leakage of dextran into the tumour vessels **(Figure 2C)**. The proportion of reduced vascular leakage was about 60% (cisplatin:5.60%; cisplatin combined with anti-IFNγ: 2.23%) **(Figure 2C)**. Finally, the GLUT1^+^ area, that reflects hypoxia in tumour tissues 37, was diminished by anti-IFNγ treatment** (Figure 2D)**, implying that anti-IFNγ might alleviated hypoxia in the tumour. Taken together, these results indicate that cisplatin-induced vascular damage is largely dependent on the increased expression of IFNγ after chemotherapy, and that neutralising IFNγ could improve vascular function.

### IFNγ promotes production of lactic acid by enhancing endothelial glycolysis

**Figure 3:**
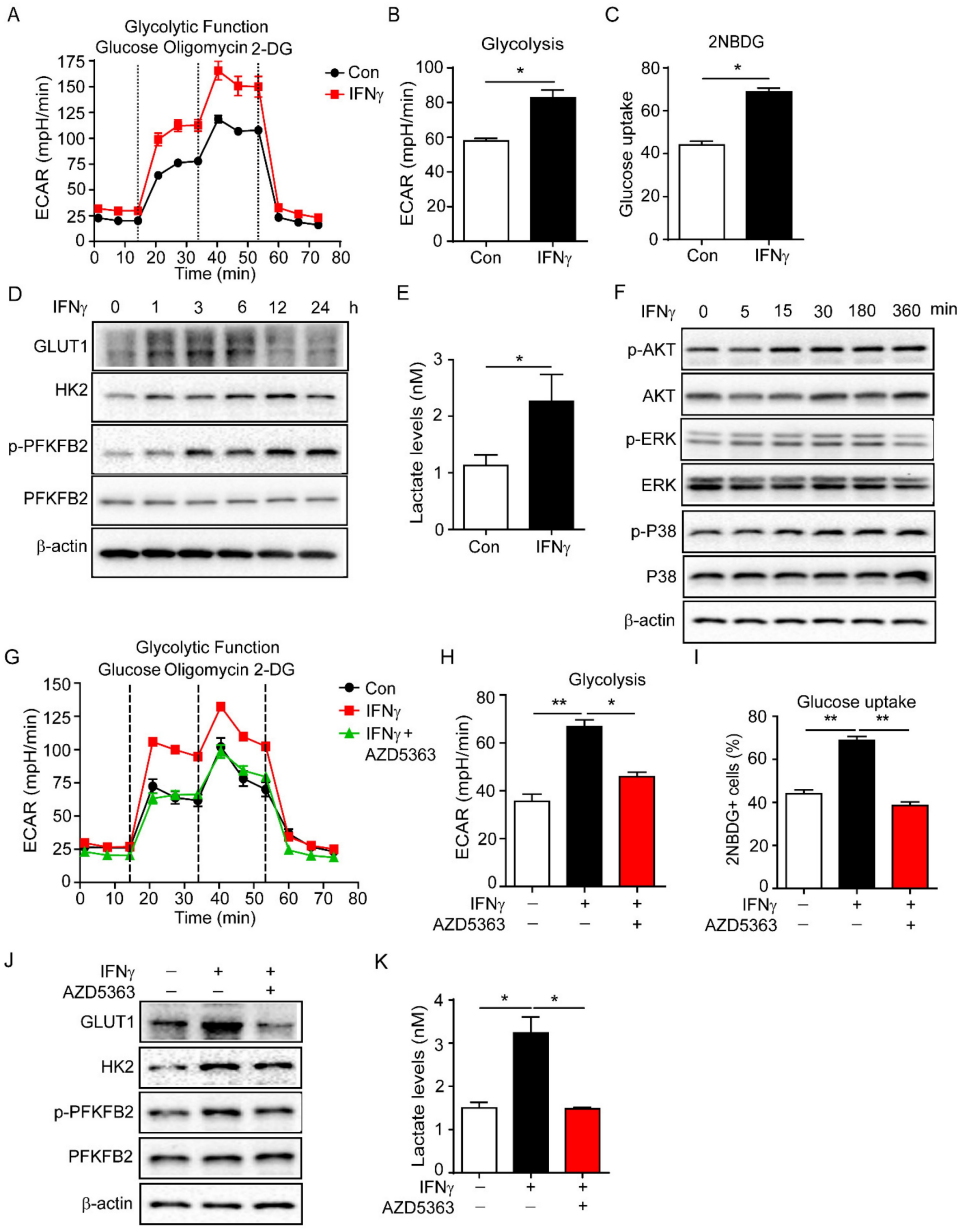
** IFNγ promotes glycolysis of endothelial cell and increases the production of lactate.** (**A**) Glycolytic function of endothelial cells stimulated with IFNγ detected by Seahorse Bioscience XF Analyser. (**B**) Statistical analysis of glycolytic function of endothelial cells with IFNγ stimulation. (**C**) Glucose uptake of endothelial cells stimulated with IFNγ measured by flow cytometry. (**D**) Protein levels of GLUT1, HK2, p-PFKFB2 and PFKFB2 in endothelial cells with IFNγ stimulation for different time. (**E**) Production of lactate by endothelial cells with IFNγ stimulation. Cellular lactate levels were normalized to protein concentration. (**F**) Activation of AKT, ERK, and P38 signalling pathways in endothelial cells with IFNγ stimulation. (**G**) Glycolysis of endothelial cells stimulated with IFNγ or IFNγ plus AKT inhibitor (AZD5363, 10 μM). (**H**) Statistical analysis of glycolysis of endothelial cells with treatment described as (**G**). (**I**) Glucose uptake of endothelial cells stimulated with IFNγ or IFNγ plus AKT inhibitor (AZD5363, 10 μM). (**J**) Protein levels of GLUT1, HK2, p-PFKFB2 and PFKFB2 in endothelial cells stimulated with IFNγ or IFNγ plus AKT inhibitor AZD5363. (**K**) Production of lactate by endothelial cells stimulated with IFNγ or IFNγ plus AKT inhibitor AZD5363. Nonparametric Mann-Whitney test. Values are mean ± SEM. *, *p* < 0.05; **, *p* < 0.01.

Endothelial cell metabolism is crucial for vascular function 4, 38. The status and function of vascular endothelial cells mainly depend on glycolysis 14, 39. Then we detected the regulation of glycolysis by IFNγ in endothelial cells. sEND.1 cell line, which was originally derived from the subcutaneous tissue of mice, was stimulated with IFNγ to detect glycolysis. Glycolysis of sEND.1 cells was increased by IFNγ **(Figure 3A, B)**.

In addition, we also detected the effect of glycolysis by IFNγ in HUVECs. The result showed that IFNγ enhanced the glycolysis of HUVECs (**Supplementary Figure S1**). Moreover, IFNγ promoted the uptake of glucose in sEND.1 cells by measuring 2-NBDG fluorescence intensity **(Figure 3C)**. Furthermore, IFNγ increased the expression of GLUT1, HK2 and PFKFB2, which are the key enzymes involved in glycolysis **(Figure 3D)**. Lactate is the final product of the glycolysis pathway and IFNγ promoted the production of lactate **(Figure 3E)**. We isolated endothelial cells from mouse tumour tissues for RNA sequencing. The results revealed that cisplatin increased the expressions of glycolysis-related genes compared with control, including PFKFB2 and HK2. Anti-IFNγ combined with cisplatin reduced the expressions of these genes compared with cisplatin alone** (Supplementary Figure S2)**. Taken together, these results demonstrated that IFNγ enhanced endothelial glycolysis and promoted the production of lactate.

To further investigate how IFNγ increased glycolysis of endothelial cells, sEND.1 cells were cultured with or without IFNγ *in vitro*. Western blotting was performed to profile the downstream signalling molecules of IFNγ. IFNγ rapidly upregulated the expression of p-AKT, p-ERK, and p-P38 **(Figure 3F)**. We then treated sEND.1 cells with IFNγ and stimulated them with or without inhibitors of AKT, ERK, and P38, respectively. First, the glycolytic function was detected by the seahorse. Results showed that IFNγ-induced increased glycolysis could be reversed by the inhibition of AKT **(Figure 3G, H)**. Inhibition of ERK and P38 did not reduce IFNγ-enhanced glycolysis (**Supplementary Figure S3**). Moreover, elevated glucose uptake induced by IFNγ was reduced by the AKT inhibitor **(Figure 3I)**. Furthermore, inhibition of AKT signalling downregulated IFNγ-promoted protein levels of GLUT1, HK2 and p-PFKFB2 **(Figure 3J)**. Lactate is the product of glycolysis, and IFNγ-induced increased lactate levels could be reduced by inhibition of AKT **(Figure 3K)**. Taken together, these results demonstrated that IFNγ increased glycolysis of endothelial cells by activating the AKT signalling pathway.

### Lactate induced by IFNγ promotes endocytosis and degradation of VE-cadherin via activating lysosome

**Figure 4:**
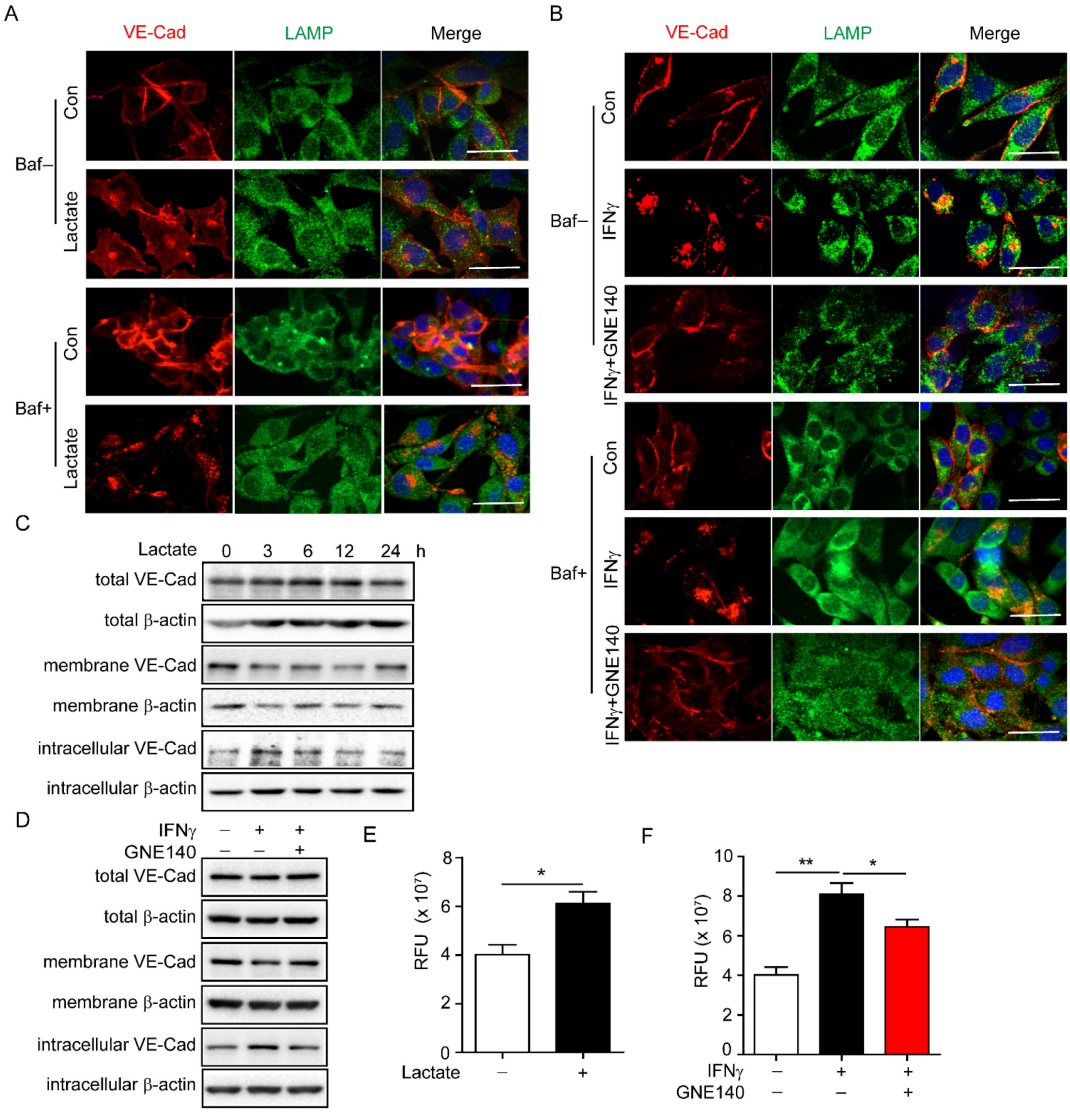
** Lactate promotes endocytosis and degradation of vascular endothelial (VE)-cadherin via activating lysosome.** (**A**) Colocalization of VE-cadherin (VE-Cad) and lysosome-associated membrane protein (LAMP) in endothelial cells with indicated treatment. Sodium lactate, 10 mΜ. Baf, bafilomycin, 100 nM. Scale bar, 20 μm. (**B**) Colocalization of VE-cadherin (VE-Cad) and LAMP in endothelial cells with indicated treatment. GNE140 (5 μM), lactate dehydrogenase A (LDHA) inhibitor. Scale bar, 20 μm. (**C**) VE-Cad expressed in total, membrane and intracellular parts of endothelial cells treated with sodium lactate for indicated time. (**D**) VE-Cad expressed in total, membrane and intracellular parts of endothelial cells treated with IFNγ and/or LDHA inhibitor GNE140. (**E**) Permeability of endothelial cell monolayers with indicated stimulations to fluorescein isothiocyanate (FITC)-dextran. Sodium lactate was used to stimulate endothelial monolayers. (**F**) Permeability of endothelial cell monolayers with indicated stimulations to FITC-dextran. IFNγ and LDHA inhibitor GNE140 were used to stimulate endothelial monolayers. Nonparametric Mann-Whitney test. Values are mean ± SEM. *, *p* < 0.05; **, *p* < 0.01.

VE-cadherin plays an important role in maintaining vascular integrity. VE-cadherin on the membrane connects gaps between endothelial cells and maintains the integrity of the blood vessels, while internalised VE-cadherin could either be recycled in the cell membrane to maintain VE-cadherin integrity or degraded in lysosomes, which leads to endothelial cell barrier dysfunction 13, 40. Lactate has been reported to enhance lysosomal activity 41. Thus, we explored whether IFNγ-induced lactic acid affected the internalisation of VE-cadherin by enhancing lysosomal activity. To facilitate the observation of the colocalization of VE-cadherin with lysosomes, indicated by lysosome-associated membrane protein (LAMP), bafilomycin A1 was added to inhibit the degradation activity of the lysosomal pathway. The immunofluorescence results revealed that lactate promoted endocytosis of VE-cadherin to lysosomes in endothelial cells **(Figure 4A)**. Inhibition of LDHA that catalyses pyruvate to lactate by GNE140 during glycolysis, reversed IFNγ-promoted endocytosis of VE-cadherin in endothelial cells **(Figure 4B)**. Moreover, lactate reduced the expression of VE-cadherin on the membrane while increasing intracellular VE-cadherin levels** (Figure 4C)**. IFNγ had the same effect on VE-cadherin; however, inhibiting LDHA diminished the effect of IFNγ **(Figure 4D)**. A permeability assay was performed to detect whether lactate promotes leakage of endothelial cells. The results showed that lactate promoted the leakage of endothelial cells **(Figure 4E)**. Inhibition of LDHA reversed the IFNγ-induced increase in the leakage of endothelial cells** (Figure 4F)**. These findings demonstrated that IFNγ-induced lactate reduced the distribution of VE-cadherin on the membrane, promoted endocytosis of VE-cadherin, and enhanced the degradation of intracellular VE-cadherin by activating lysosomes.

### Properly tuning IFNγ responses repairs vascular damage and increases drug delivery

**Figure 5:**
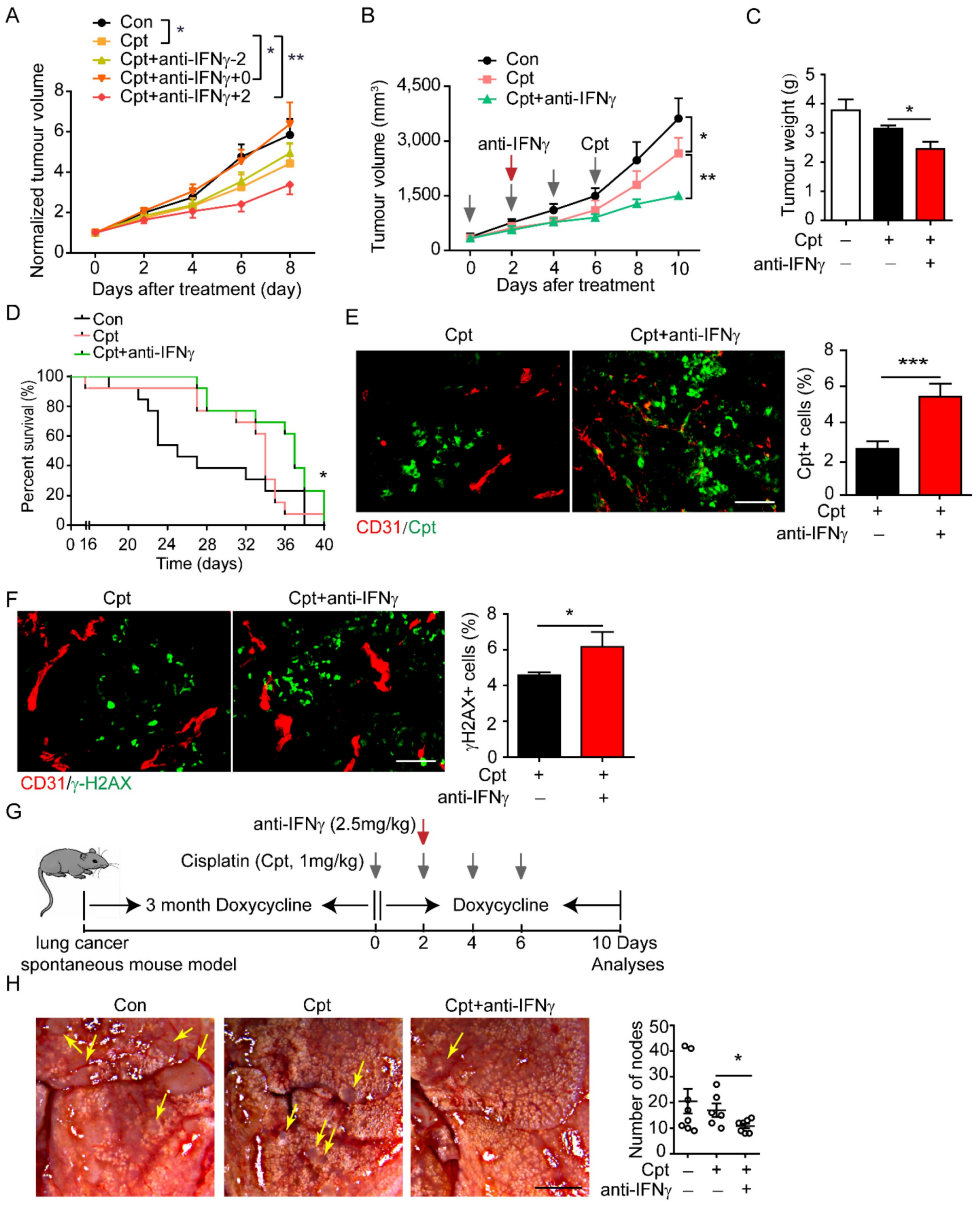
** Screening time window of neutralizing IFNγ for the improvement of drug delivery and chemotherapy.** (**A**) Tumour growth of Lewis lung carcinoma (LLC) tumours treated with indicated drugs. Cisplatin (Cpt, 1 mg/kg) and anti-IFNγ antibody (2.5 mg/kg) were applied on the same day (Cpt+anti-IFNγ+0), two days before the first Cpt injection (Cpt+anti-IFNγ-2) or two days after the first Cpt injection (Cpt+anti-IFNγ+2). Tumour volumes were normalized to that of each mouse at day 0 when Cpt was applied. n = 8 for phosphate buffered saline, Cpt. n = 7 for Cpt+anti-IFNγ+0, Cpt+ anti-IFNγ-2. n = 5 for Cpt+ anti-IFNγ+2. Two-way ANOVA test. *, *p* < 0.05; **, *p* < 0.01. (**B**) Tumour growth of LLC tumours treated with indicated drugs. Two-way ANOVA test. *, *p* < 0.05; **,* p* < 0.01. (**C**) Tumour weight of LLC tumours treated with indicated drugs on 10 days after treatment. (**D**) Survival curves of LLC tumour-bearing mice treated with indicated drugs. n = 9-12 for each group. Log-rank test. *, *p* < 0.05. (**E**) Representative images and quantifications of Cpt-DNA adducts in tumours treated with Cpt or Cpt+anti-IFNγ. Tumours were harvest eight hours after administration of a single dose of Cpt (10 mg/kg) on day 20 after tumour transplantation. Scale bar, 100 μm. (**F**) Representative images and quantifications of DNA damages (indicated by γ-H2AX staining) in tumours treated with Cpt or Cpt+anti-IFNγ. Tumours were harvested on day 22 after tumour transplantation. Scale bar, 100 μm. (**G**) Diagram depicting generation of mouse spontaneous lung cancer model (TetO-EGFR^L858R^; CCSP-rtTA) and treatment schedule. (**H**) Representative images and quantifications of lung cancer nodes from mice with treatment described in (**G**). Arrows, tumour nodes. Scale bar, 2 mm. Results showing are data collected from two experiments. Unless otherwise denoted, nonparametric Mann-Whitney test was used. Values are mean ± SEM. *, *p* < 0.05; ***, *p* < 0.001.

Destruction of tumour blood vessels reduces the osmotic concentration of chemotherapeutic drugs in the tumour tissue, thereby weakening the effect of killing tumour cell, leading to chemotherapy resistance 42. In view of the observation that neutralising IFNγ blocked the destruction of tumour blood vessels by cisplatin, we speculated that neutralising IFNγ might promote drug delivery. Considering that IFNγ is a key mediator of anti-tumour immunity, we applied an anti-IFNγ antibody at different time points to screen the time window for beneficial outcomes. The application of cisplatin (1 mg/kg) significantly inhibited tumour growth **(Figure 5A).** When anti-IFNγ antibody was administered two days before the first injection of cisplatin, it did not affect the outcome of chemotherapy **(Figure 5A)**. However, when anti-IFNγ antibody was administered simultaneously with cisplatin, it impaired the beneficial effects of chemotherapy **(Figure 5A)**, which was consistent with the observation that IFNγ might be involved in anti-tumour immunity during chemotherapy. When anti-IFNγ antibody was administered two days after the first injection of cisplatin, it largely promoted tumour chemotherapy **(Figure 5A, B)**.

We further explored effects of neutralising IFNγ after the first cisplatin injection. Neutralising IFNγ combined with cisplatin reduced tumour volume **(Figure 5B)** and tumour weight **(Figure 5C)** to a greater degree compared than that reduced by cisplatin alone. Moreover, neutralising IFNγ combined with cisplatin significantly increased the overall survival of mice compared that increased by cisplatin alone **(Figure 5D)**. Cpt-DNA adducts within tumour tissues were increased by anti-IFNγ treatment and the effective rate of drug delivery was increased by about 113% (cisplatin: 2.55%; cisplatin combined with anti-IFNγ: 5.44%) **(Figure 5E)**, as shown by immunostaining. Cisplatin causes DNA cross-linking and stimulates H2AX phosphorylation at serine 139 to generate γ-H2AX as a major marker for DNA damage signalling in response to DNA double-strand breaks 43. Moreover, γ-H2AX^+^ area in the tumour tissue was increased by neutralising IFNγ treatment **(Figure 5F)**. These results indicated that neutralising IFNγ enhanced the delivery of cisplatin into the tumour, thereby promoting the chemotherapeutic effect.

In addition to the lung cancer xenograft mouse model, we constructed a lung cancer spontaneous mouse model (TetO-EGFR^L858R^; CCSP-rtTA). After sustained induction by doxycycline (2 mg/mL) for three months, cisplatin was injected every other day for four times. The IFNγ neutralising antibody was given two days after the first cisplatin injection. During this period, doxycycline was continually administered **(Figure 5G)**. First, pericyte coverage was detected by examining the expression of NG2^+^CD146^+^ tumour blood vessels by immunofluorescence analysis and found that anti-IFNγ increases NG2^+^ pericyte coverage compared with that observed in cisplatin-treated tumours (**Supplementary Figure S4**), thus implying that compared with cisplatin, anti-IFNγ enhances the integrity of tumour blood vessels. Then tumour nodes in the lungs were counted. In this model, cisplatin treatment did not significantly reduce the number of tumour nodes in the lung compared with that in the control. Neutralisation of IFNγ combined with cisplatin significantly reduced the number of lung nodes compared with that by cisplatin treatment alone **(Figure 5H)**.

### IFNγ blockade in capillary leak site prevents cisplatin-induced vascular damages and improves drug delivery

**Figure 6:**
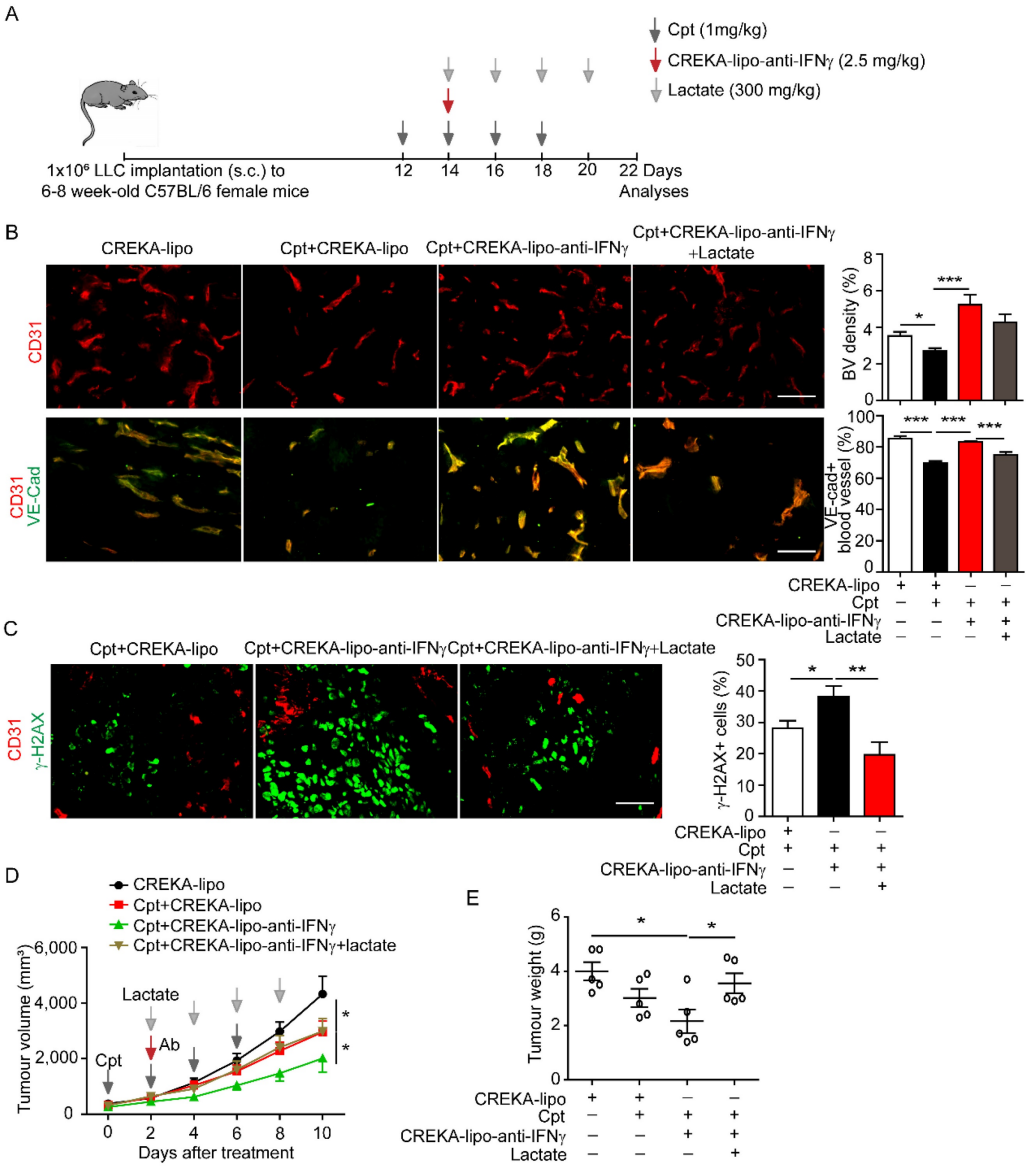
** Delivery of anti-IFNγ antibody to capillary leak site by CREKA-lipo-anti-IFNγ nanoparticles repairs cisplatin-induced vascular damages and improves drug penetration.** (**A**) Diagram depicting generation of Lewis lung carcinoma (LLC) tumour model and treatment schedule. Cisplatin (Cpt, 1 mg/kg). CREKA-lipo-anti-IFNγ antibody, 2.5 mg/kg. Lactate, 300 mg/kg. (**B**) Representative images and quantifications of vascular density and vascular expression of junctional protein VE-cadherin (VE-Cad) in tumour tissues with indicated treatment. Scale bar, 100 μm. (**C**) Representative images and quantifications of DNA damages (indicated by γ-H2AX staining) in tumour tissues with indicated treatment. Mice were sacrificed on day 22. Scale bar, 100 μm. (**D-E**) Tumour growth and tumour weight of LLC tumours with indicated treatment. Treatment started from 12 days after implantation. Black arrows, Cpt (1 mg/kg) injection., Red arrow, CREKA-lipo or CREKA-lipo-anti-IFNγ antibody (2.5 mg/kg) injection. Grey arrows, lactate (300 mg/kg) injection. Two-way ANOVA test. *, *p* < 0.05. Unless otherwise denoted, Nonparametric Mann-Whitney test was used. Values are mean ± SEM. *, *p* < 0.05; ***, *p* < 0.001.

**Figure 7:**
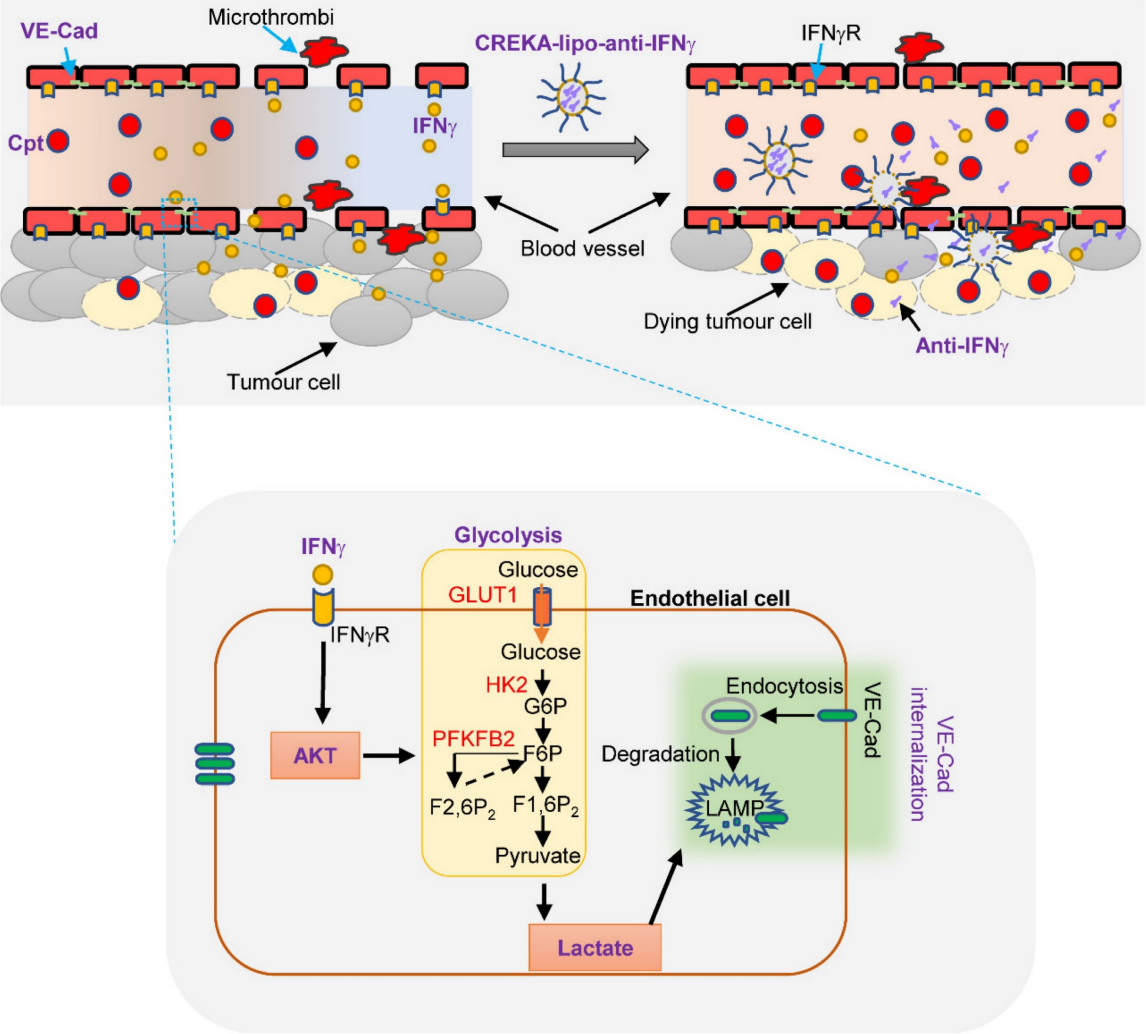
** Diagram depicting IFNγ blockade in capillary leak site improves chemotherapy of tumour via inhibiting lactate-induced VE-cadherin's endocytosis.** Cisplatin treatment induces the increase of IFNγ, which promotes the endocytosis of VE-cadherin (VE-Cad) by AKT-dependent instigation of endothelial glycolysis to produce lactate. CREKA-coated nanoparticles carrying anti-IFNγ antibodies (CREKA-lipo-anti-IFNγ) locally blocks to a capillary leak site by specifically targeting the microthrombi on the walls of leaky blood vessels. Neutralisation of IFNγ prevents vascular damages and enhances cisplatin efficacy in cancer treatment.

The outcome of systematic application of anti-IFNγ antibodies is difficult to control because of various targets. We sought to locally deliver anti-IFNγ antibodies to the capillary leak site during chemotherapy. We coated nanoparticles with CREKA and loaded anti-IFNγ antibodies into nanoparticles (CREKA-lipo-anti-IFNγ). The tumour-homing pentapeptide (CREKA) on the surface of nanoparticles specifically targets fibrin-fibronectin complexes within the microthrombi that are found on the walls of leaky tumour blood vessels. We confirmed that the tumour blood vessels were enwrapped by fibronectin in the LLC tumour with cisplatin treatment (**Supplementary Figure S5**). Then the characterization detection of CREKA-lipo-anti-IFNγ was performed. We have determined the diameter, potential and release profiles of the CREKA-lipo-anti-IFNγ nanoparticle. Dynamic light scattering analysis showed that the average hydrodynamic diameter of CREKA-Lipo-anti-IFNγ was about 316 nm, which is larger than the diameter of empty nanoparticles (CREKA-Lipo; 245 nm) (**Supplementary Figure S6A-B**). In response to the addition of anti-IFNγ, the average zeta potential of the particles increased from -0.675 mV to -0.275 mV (**Supplementary Figure S6C**), thereby indicating that drug loading affected the net charge of the nanoparticles. To evaluate the drug release profiles of CREKA-Lipo-anti-IFNγ, the accumulative drug release at both pH 6.5 and 7.4 was investigated. The result showed that CREKA-Lipo-anti-IFNγ is characterized by a relatively more rapid drug release at pH 6.5 than at pH 7.4. At pH 6.5, we detected approximately 43% accumulative drug release at 48 h (**Supplementary Figure S6D**). Because acidic condition enhances the release of drugs by CREKA-lipo nanoparticles 8, 31, when CREKA-lipo-anti-IFNγ was delivered into the tumour, the tumour acidic environment will elicit the local release of anti-IFNγ antibodies by the nanoparticles. We also evaluated the biosafety of CREKA-lipo-anti-IFNγ nanoparticles, we did not observe apparent toxicity of the nanoparticles to major organs (**Supplementary Figure S7A, B**), in consistence with previous study 31. For comparison, lactate was intravenously administered in mice two days after the first injection of cisplatin, once every other day, for a total of four times **(Figure 6A)**. We found that CREKA-lipo-anti-IFNγ combined with cisplatin treatment increased the density of CD31^+^ tumour blood vessels and the distribution of endothelial junctional molecule VE-cadherin when compared with those by cisplatin alone, while lactate reversed the effects of CREKA-lipo-anti-IFNγ on blood vessels **(Figure 6B)**. Moreover, DNA damage indicated by γ-H2AX staining in tumour tissues was increased by CREKA-lipo-anti-IFNγ treatment, which was inhibited by lactate treatment **(Figure 6C)**. CREKA-lipo-anti-IFNγ combined with cisplatin reduced tumour volume compared with those reduced by cisplatin alone, while lactate abrogated the effect of CREKA-lipo-anti-IFNγ **(Figure 6D)**. CREKA-lipo-anti-IFNγ combined with cisplatin reduces tumour weight compared with that in control group mice, whereas lactate abrogates the effect of CREKA-lipo-anti-IFNγ **(Figure 6E)**. In summary, these results demonstrated that the delivery of IFNγ blockade to the capillary leak site prevented cisplatin-induced vascular damage, thereby promoting drug delivery and improving the chemotherapy effect **(Figure 7)**.

## Discussion

IFNγ is believed to be a central mediator of anti-tumour immunity 20; however, many studies have demonstrated controversial roles of IFNγ in tumour progression 44. It is possible that advanced tumours escape anti-tumour immunity and hijack immune responses to accelerate tumour progression 20. Alternatively, IFNγ may aggravate the lethal side effects of chemotherapeutic drugs 36. From a translational point of view, successful strategies to modulate IFNγ for tumour therapy are still lacking 45. In this study, we demonstrated that the delivery of anti-IFNγ antibody to the capillary leaky site prevented cisplatin-induced vascular damage, thereby promoting drug delivery and improving tumour chemotherapy. Cisplatin treatment induces an increase in IFNγ, which promotes endocytosis of VE-cadherin by inducing endothelial glycolysis and lactate production. Neutralising IFNγ maintained VE-cadherin on the endothelial membrane and guaranteed vascular integrity, providing an efficient tunnel for drug delivery. Therefore, these results provide a basis for the tissue-specific IFNγ blockade for enhancing tumour chemotherapy.

Inhibition of IFNγ around leaky blood vessels is an effective way to promote vascular integrity and drug penetration in tumour 8. VE-cadherin on endothelial cells is important for the maintenance of vascular integrity, and its loss causes vascular leakage 13. We found that chemotherapy treatment induced IFNγ-dependent loss of VE-cadherin on endothelial cells and increased vascular permeability, consistent with previous studies that show that chemotherapeutic drugs cause capillary leakage 6. At first, we identified a time window when the application of anti-IFNγ antibody shortly after cisplatin promoted chemotherapy, while other time points did not. We were also surprised when we observed this response, which is an interesting phenomenon and warrants further study. IFNγ is a cytokine that has both protumour and antitumour activities, which are determined by differences in tumour microenvironments and stages, as well as the different targets of IFNγ 23, 46. We observed that anti-IFNγ antibodies administered two days after the first injection of cisplatin can promote tumour chemotherapy. Cisplatin treatment was found to promote an increase in the expression of IFNγ, enhance endothelial cell glycolysis and lactic acid production, promote VE-cadherin endocytosis, and promote the disruption of tumour vascular integrity. Furthermore, the injection of IFNγ neutralizing antibodies two days after the first injection of cisplatin was observed to neutralize the increase in IFNγ promoted by cisplatin, reverse IFNγ-induced tumour blood vessels damage, enhance the integrity of tumour blood vessels, and promote an increase in the delivery of cisplatin into tumour tissues. In contrast, however, anti-IFNγ administered two days prior the first injection of cisplatin failed to promote chemotherapy. At this point, the tumour tissue was still small and at a relatively early stage, and at this stage, it is plausible that IFNγ may be mainly effective in killing tumour cells rather than regulating blood vessels. Moreover, neutralizing IFNγ was found to reduce the anti-tumour effects. When anti-IFNγ antibodies were administered simultaneously with cisplatin, tumour volume was observed to be similar to that in the control group. Given that IFNγ has been shown to inhibit the fibroblast-diminished nuclear accumulation of platinum via altering glutathione and cysteine metabolism in fibroblasts 47, we speculate that the simultaneous injection of anti-IFNγ and cisplatin may inhibit the entry of cisplatin into tumour cells or promote cisplatin pumping, thereby inducing a resistance to chemotherapy. Considering the increasing recognition of the dual role played by IFNγ in tumour development, it will be necessary to gain a more comprehensive understanding of the effects of IFNγ in the future. Proper application of anti-IFNγ increased Cpt-DNA adducts in tumour tissues, indicating the promotion of drug delivery. Nevertheless, the outcome of the systematic application of IFNγ antibody is difficult to control because of various targets. The tumour-homing pentapeptide (CREKA) targets the fibrin-fibronectin complex that appears in tumour capillary leaky sites 8, 31. CREKA is a peptide with specific affinity for clotted plasma proteins as the targeting element. The interstitial spaces of tumours contain fibrin and proteins that become cross-linked to fibrin in blood clotting, such as fibronectin. The presence of these products in tumours, but not in normal tissues, is thought to be a result of leakiness of tumour vessels. The intravascular clots further attract more nanoparticles into the tumour, amplifying the targeting (7). In this study, CREKA-coated nanoparticles CREKA-lipo-anti-IFNγ were generated. The application of CREKA-lipo-anti-IFNγ successfully repaired vascular integrity, improved drug delivery, and enhanced chemotherapy, which provides the possibility for clinical application. Additionally, by loading probes into the CREKA-lipo nanoparticles, the nanomaterial might be used to diagnose the degree of vascular damages in tumour chemotherapy and inform the chances for application of IFNγ neutralizing antibody. In contrast to our findings, it has been reported that blocking immune checkpoint activates CD4^+^ T lymphocytes or CD8^+^ T lymphocytes to produce IFNγ, thereby increasing tumour blood vessel normalisation 48, 49. This discrepancy might be due to the distinct tumour microenvironment. It is possible that IFNγ mainly inhibits hyperproliferative sprouting vasculatures in blocking immune checkpoint, while IFNγ stimulates glycolytic endothelial cells to accelerate the disruption of vascular integrity in tumours with chemotherapy. Besides, anti-PD-1 treatment increases the expression of IFNγ, which mediates tumor immune escape by enhancing the expression of immunosuppressive molecules, such as IDO-1 and PD-L1^46^, ^50^. In our study, cisplatin induced expression of IFNγ, which might promote anti-PD-1 resistance. Anti-IFNγ combined with anti-PD-1 therapy and chemotherapy might promote anti-tumor effects further. Although we demonstrate the possibility of application of anti-IFNγ antibody to restore vascular integrity for drug delivery in this study, the neutralising anti-IFNγ antibody might have dosage-dependent effects on the treatment of tumour that needs further studies in different treatment conditions.

IFNγ-induced internalisation of VE-cadherin is a major mechanism of cisplatin-induced vascular leakage in tumours. Inflammatory cytokines play an important role in chemotherapy-induced vascular damage in tumours. These results revealed that tumour blood vessels were damaged after cisplatin treatment. Tumour cells are more sensitive than endothelial cells to cisplatin treatment 51. The findings of this study also revealed that cisplatin-DNA adducts were mainly located in tumour cells with few located in vascular endothelial cells. These results suggest that cisplatin may affect tumour blood vessels through an indirect mechanism. In contrast to some conventional chemotherapeutic agents that are immunosuppressive, cisplatin can stimulate anti-tumour immune responses 35. Our results revealed that the expression of IFNγ was increased after cisplatin treatment. Kammertoens et al. also reported that doxorubicin induced the expression of IFNγ, which in turn caused tumour vascular depression 21. Previous studies have identified that IFNγ plays an important role in the regulation of tumour blood vessels 17-19, 26. In this study, results revealed that IFNγ stimulation induced the endocytosis of VE-cadherin, neutralised IFNγ reversed cisplatin-induced vascular damage, improved the expression of VE-cadherin on tumour blood vessels, and promoted tumour vascular functions. Therefore, cisplatin largely destroys tumour blood vessels by stimulating IFNγ production. Admittedly, cisplatin-induced other inflammatory molecules may also play partial roles, such as TNFα and IL-1β. Indeed, TNFα has also been shown to inhibit tumour angiogenesis via TNFR2 and specific blockade of TNFα slows blood vessel remodeling 52, 53. It has also been established that IL-1β can promote angiogenesis, suppress VE-cadherin transcription and destabilize adherens junctions in blood vessels 54, 55. We anticipate that the roles of TNFα and IL-1β in cisplatin-induced tumour vascular damage will be further investigated in the future.

IFNγ stimulates the internalisation of VE-cadherin in endothelial cells by modulating endothelial glycolysis. Our findings showed that the IFNγ-stimulated loss of VE-cadherin on endothelial cells was dependent on lactate production. IFNγ increased endothelial glycolysis and lactate production by activating AKT signalling. AKT is a signalling pathway that primarily promotes glycolysis 56. Consistent with our results, AKT has been reported to modulate the activity of hexokinases 57. Lactate has been reported to increase the density of lysosomes and support lysosomal acidification, thus enhancing the degradation of proteins in the lysosomal-dependent pathway 41. Internalised VE-cadherin can be recycled to the cell membrane to maintain endothelial integrity or degraded in lysosomes, leading to vascular dysfunction 13, 40. Our findings demonstrated that lactate increased endocytosis and degradation of VE-cadherin, leading to hyperpermeability. Consistent with our findings, proto-oncogene tyrosine-protein kinase SRC (SRC) has been reported to be a key regulator of VE-cadherin internalisation, and IFNγ could increase SRC activity 40, 58. SRC has also been linked to the activation of glycolysis 59. Tyrosine phosphorylation of the adhesion molecule VE-cadherin is assumed to affect endothelial junction integrity. Wessel et al. has demonstrated that Tyr685 phosphorylation of VE-cadherin regulates the induction of vascular permeability 60. However, whether IFNγ and lactate modulate Tyr685 phosphorylation of VE-cadherin requires further study. Collectively, we conclude that the integrity of endothelial cells in tumours with chemotherapy is controlled by IFNγ-stimulated glycolysis.

IFNγ blockade has the potential to prevent chemotherapy-associated lethal side effects. Chemotherapy treatment-associated side effects are a cause of death in patients with cancer. In cancer patients, chemotherapeutic drugs have been reported to cause capillary leak syndrome 6. Chemotherapy (including cisplatin) is a strong inducer of thrombosis and is an independent risk factor for the development of venous thromboembolism, a lethal disease 61. The absence of IFNγ has been reported to accelerate thrombus resolution 62. Therefore, blocking IFNγ by CREKA-lipo-anti-IFNγ has the potential to prevent capillary leak syndrome and venous thromboembolism in cancer patients undergoing chemotherapy, which merits further studies. Our previous study demonstrated that blocking IFNγ was an efficient way to prevent doxorubicin-induced cardiotoxicity 36, which supports the application of anti-IFNγ antibody for the benefits of cancer patients. Together, our results demonstrate that the delivery of anti-IFNγ antibody to the capillary site by CREKA-lipo-anti-IFNγ may be beneficial for cancer patients undergoing chemotherapy, which can be explored as a new strategy for the treatment of lung cancer.

## Conclusion

In summary, we demonstrate that IFNγ blockade in capillary leak site prevents drug-induced vascular damages, improves drug delivery and chemotherapeutic efficacy in mouse lung cancer models. Cisplatin treatment induces the increase of IFNγ, which promotes the endocytosis of VE-cadherin through instigating endothelial glycolysis and lactate production. Neutralizing IFNγ maintains VE-cadherin on endothelial membrane and guarantees vascular integrity, providing an efficient tunnel for drug delivery. Our results provide a basis for tissue-specific delivery of IFNγ blockade for the improvement of tumor chemotherapy.

## Supplementary Material

Supplementary figures.

## Abbreviations

2-NBDG: 2-[N-(7-Nitrobenz-2-oxa-1,3-diaxol-4-yl)amino]-2-deoxyglucose
ALT: alanine aminotransferase
AST: aspartate aminotransferase
CBA: Cytometric Bead Array
CPT: cisplatin, [cis-diamminedichloridoplatinum (II)]
CR: creatinine
DOPC: dioleoylphosphatidylcholine
ECAR: extracellular acidification rate
FBS: fetal bovine serum
FITC: fluorescein isothiocyanate
LAMP: lysosome-associated membrane protein
LLC: Lewis lung carcinoma
PFA: paraformaldehyde
SRC: proto-oncogene tyrosine-protein kinase SRC
VE-cadherin: vascular endothelial cadherin
WT: wild-type
NG2: neuron-glial antigen 2
PBS: phosphate buffered saline
DLS: dynamic light scattering
