# Supplementary material for: IFNγ blockade in capillary leak site improves tumour chemotherapy by inhibiting lactate-induced endocytosis of vascular endothelial-cadherins
Source: Int J Biol Sci. 2023 Feb 27;19(5):1490–508. doi: 10.7150/ijbs.78248 (PMC10086745; doi:10.7150/ijbs.78248)
Supplement: Supplementary file 1 — Supplementary figures. [file ijbsv19p1490s1.pdf]

Supplemental Figures and Figure Legends

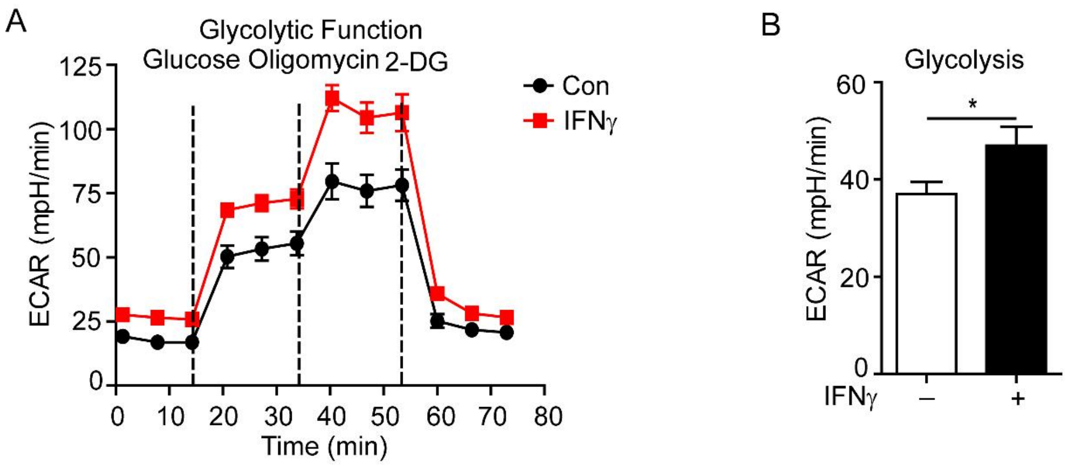

Figure S1. IFNγ enhanced glycolysis of HUVECs. (**A**) Glycolysis of HUVECs (3000 cells per well) stimulated with IFNγ (30 ng/mL) . (**B**) Statistical analysis of glycolytic function of HUVECs with IFNγ stimulation. Statistical analyses using Nonparametric Mann-Whitney test. Values are mean ± SEM. *, $p < 0.05$;

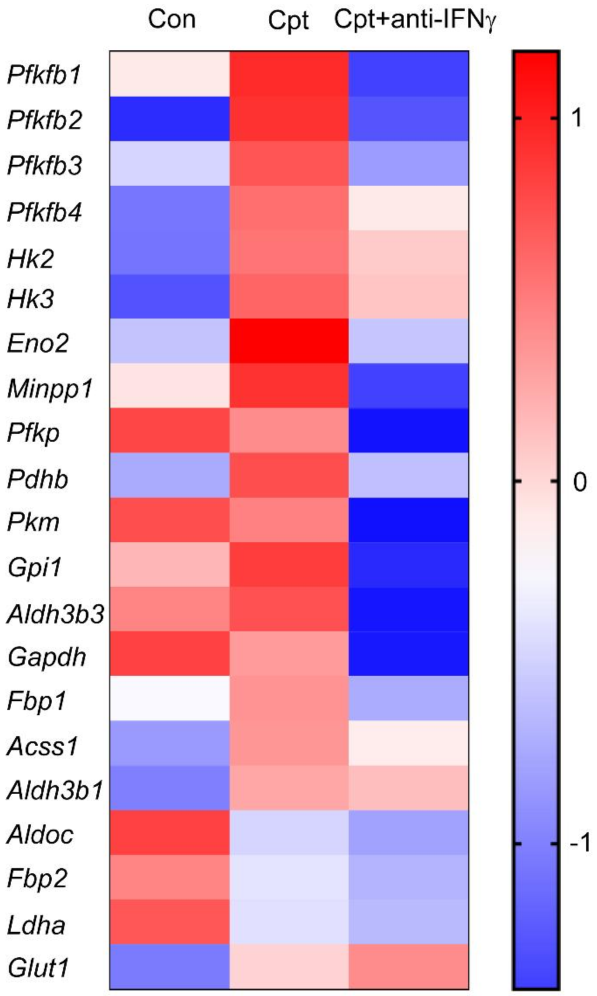

Figure S2. Heatmap of transcript levels of genes in glycolysis. The expressions of glycolysis-related genes of isolated tumour vascular endothelial cells from mouse tumour tissues with indicated treatments. Statistical analyses using Z-score normalization. N=3 for each group. Color scale: red, high expression; blue, low expression.

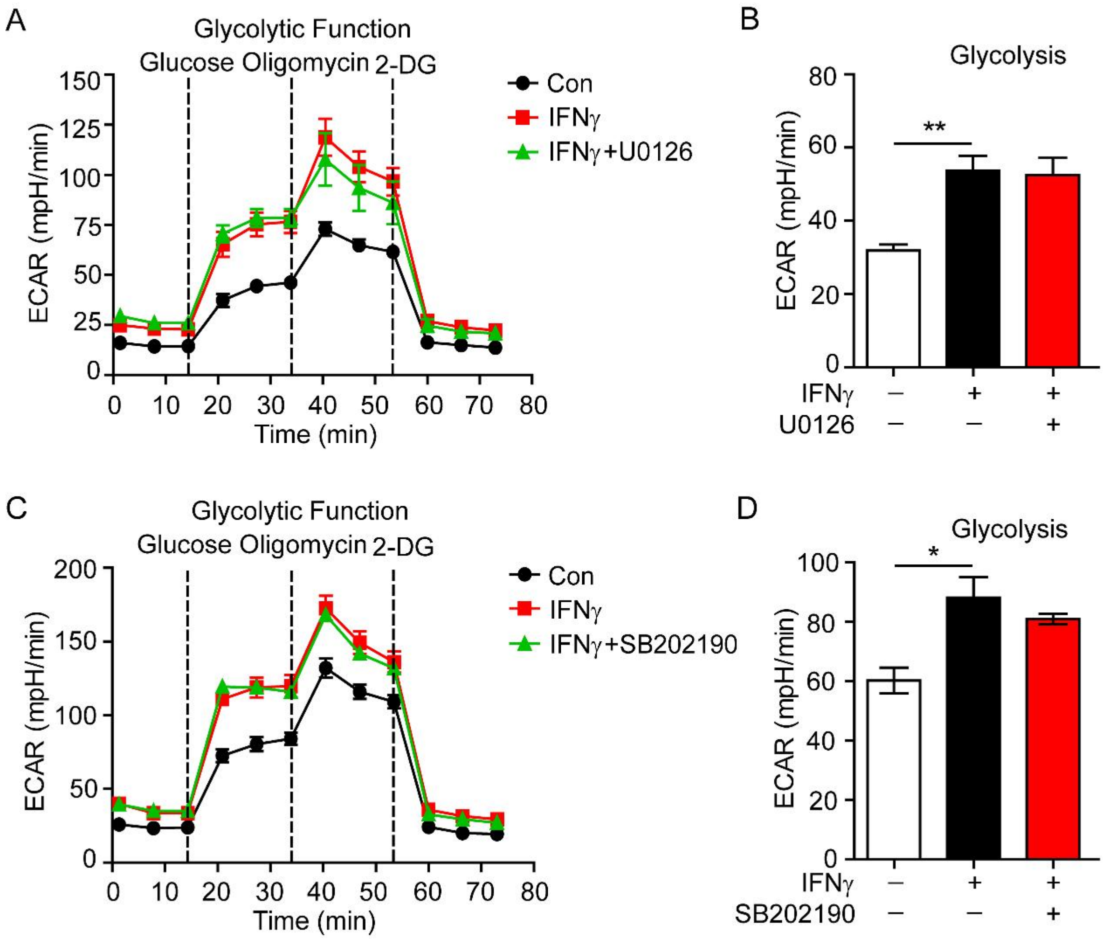

Figure S3. Inhibition of ERK and P38 do not affect IFNγ-stimulated glycolysis of endothelial cells. (**A**) Glycolysis of endothelial cells stimulated with IFNγ or IFNγ plus ERK inhibitor (U0126, 10 μM). (**B**) Statistical analysis of glycolysis of endothelial cells with treatment described as (**A**). Statistical analyses using Nonparametric Mann-Whitney test. Values are mean ± SEM. **, $p< 0.01$; (**C**) Glycolysis of endothelial cells stimulated with IFNγ or IFNγ plus P38 inhibitor (SB202190, 10 μM). (**D**) Statistical analysis of glycolysis of endothelial cells with treatment described as (**C**). Statistical analyses using Nonparametric Mann-Whitney test. Values are mean ± SEM. *, $p < 0.05$.

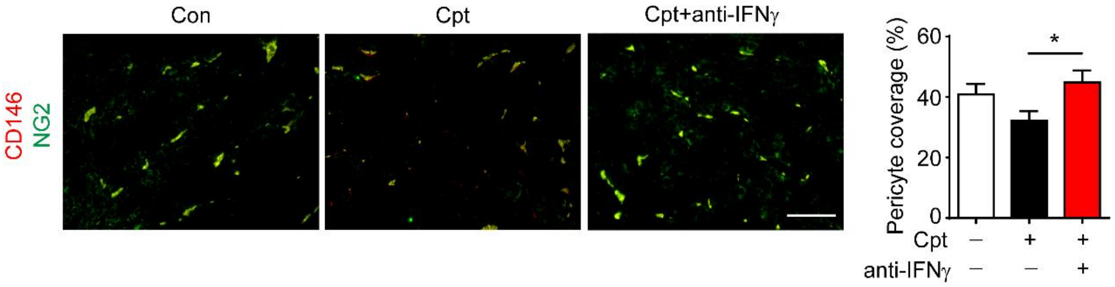

Figure S4. Neutralizing IFNγ reversed cisplatin-reduced pericyte coverage. Representative images and quantifications of pericyte coverage in tumour tissues with different treatments. Scale bar, 100 μm. Nonparametric Mann-Whitney test. Values are mean ± SEM. *, $p < 0.05$.

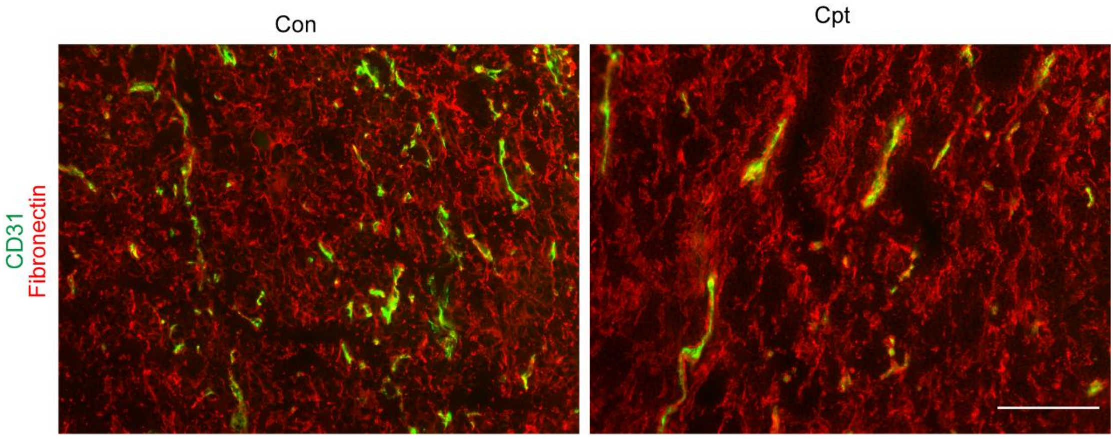

Figure S5. Tumour blood vessels are enwrapped by fibronectin in LLC tumour with cisplatin treatment. Co-staining of CD31 and fibronectin in tumour sections from control (Con) or cisplatin (Cpt) treated LLC tumours. Scale bar, 100 μm.

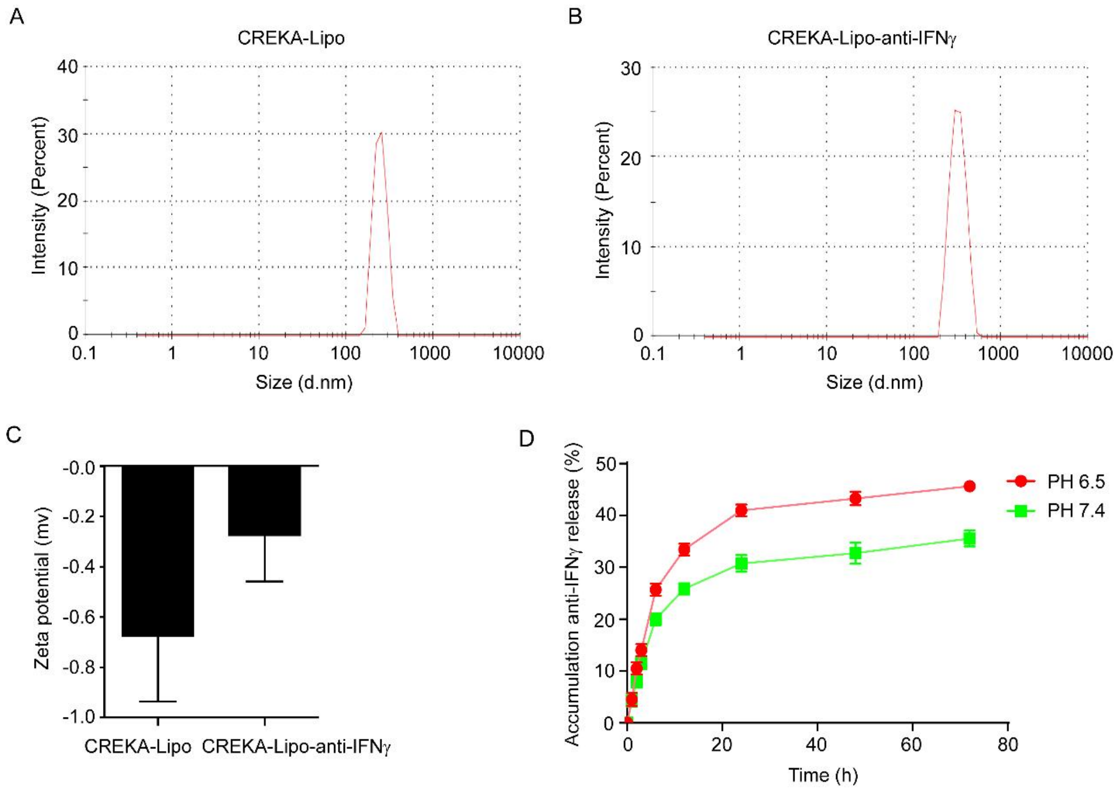

Figure S6. Characterization and release profiles of the CREKA-lipo-anti-IFNγ nanoparticle. (A-C) Size distribution and zeta potential characterization of CREKA-lipo and CREKA-lipo-anti-IFNγ at (Day 0) (n=3) in the PBS buffer at room temperature. The data of zeta potential are presented as the mean ± standard deviation (n=3). **(D)** The drug release profiles of CREKA-Lipo-anti-IFNγ at two pH conditions. The data are presented as the mean ± standard deviation (n=3).

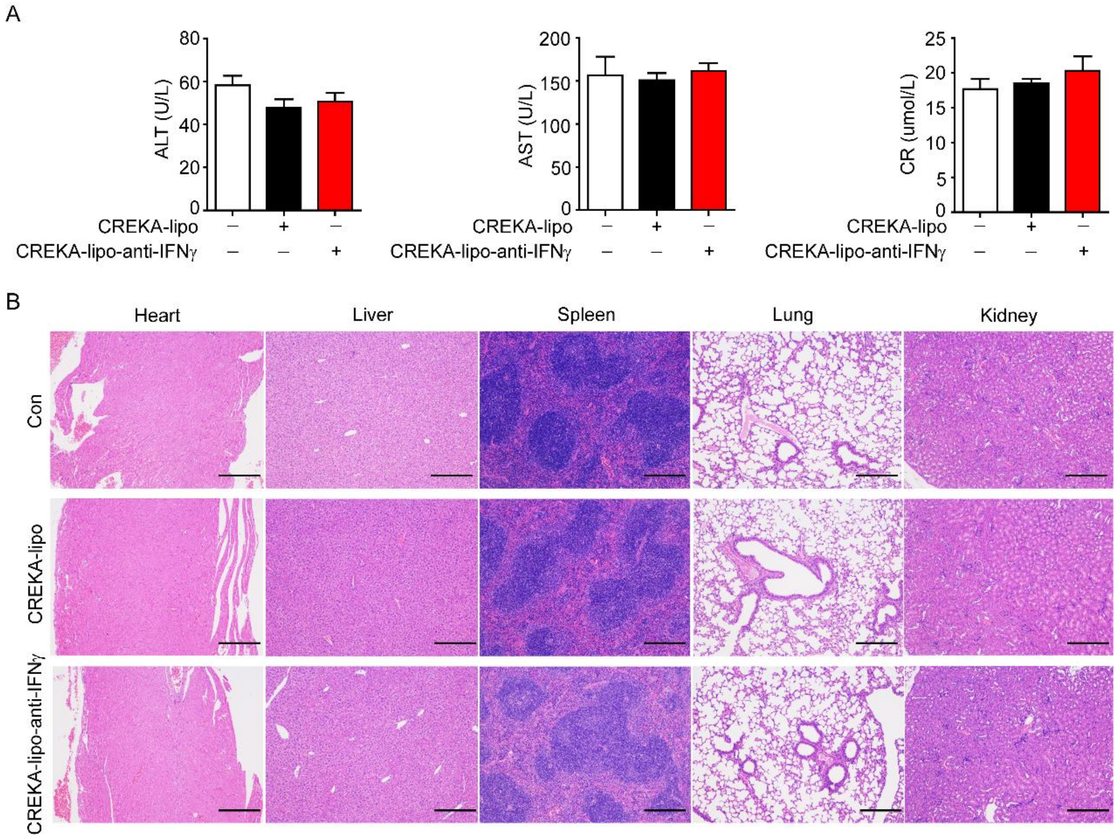

Figure S7. Evaluation of the biosafety of CREKA-lipo-anti-IFNγ *in vivo*. (A) The effects of CREKA-lipo-anti-IFNγ on the serum level of aspartate aminotransferase (AST), alanine aminotransferase (ALT) related to liver function, and creatinine (CR) related to kidney function. Statistical analyses using nonparametric Mann-Whitney test (n = 5-6 for each group). (**B**) CREKA-lipo-anti-IFNγ treatment showed no visible damage to the major organs of mice as indicated by H&E staining (n = 3 for each group). Scale bar, 200 μm.
